# Consensus clustering approach to group brain connectivity matrices

**DOI:** 10.1162/NETN_a_00017

**Published:** 2017-10-01

**Authors:** Javier Rasero, Mario Pellicoro, Leonardo Angelini, Jesus M. Cortes, Daniele Marinazzo, Sebastiano Stramaglia

**Affiliations:** Biocruces Health Research Institute. Hospital Universitario de Cruces, Barakaldo, Spain; Dipartimento di Fisica, Università degli Studi Aldo Moro, Bari, Italy; Istituto Nazionale di Fisica Nucleare, Sezione di Bari, Italy; TIRES-Center of Innovative Technologies for Signal Detection and Processing, Università degli Studi Aldo Moro Bari, Italy; Ikerbasque, the Basque Foundation for Science, Bilbao, Spain; Faculty of Psychology and Educational Sciences, Department of Data Analysis, Ghent University, Ghent, Belgium

**Keywords:** Unsupervised learning, Consensus clustering, Resting fMRI, Structural DTI

## Abstract

A novel approach rooted on the notion of *consensus* clustering, a strategy developed for community detection in complex networks, is proposed to cope with the heterogeneity that characterizes connectivity matrices in health and disease. The method can be summarized as follows: (a) define, for each node, a distance matrix for the set of subjects by comparing the connectivity pattern of that node in all pairs of subjects; (b) cluster the distance matrix for each node; (c) build the consensus network from the corresponding partitions; and (d) extract groups of subjects by finding the communities of the consensus network thus obtained. Different from the previous implementations of consensus clustering, we thus propose to use the consensus strategy to combine the information arising from the connectivity patterns of each node. The proposed approach may be seen either as an exploratory technique or as an unsupervised pretraining step to help the subsequent construction of a supervised classifier. Applications on a toy model and two real datasets show the effectiveness of the proposed methodology, which represents heterogeneity of a set of subjects in terms of a weighted network, the consensus matrix.

## INTRODUCTION

In the supervised analysis of human connectome data (Craddock et al., 2013; Sporns, 2010), subjects are usually grouped under a common umbrella corresponding to high-level clinical categories (e.g., patients and controls), and typical approaches aim at deducing a decision function from the labeled training data (see Fornito & Bullmore, 2010). However, the populations of subjects (healthy as well as patients) are usually highly heterogeneous: clustering algorithms find natural groupings in the data, and therefore constitute a promising technique for disentangling the heterogeneity that is inherent to many conditions, and to the cohort of controls. Such an unsupervised classification may also be used as a preprocessing stage, so that the subsequent supervised analysis might exploit the knowledge of the structure of data. Some studies dealt with similar issues: semisupervised clustering of imaging data was considered in Filipovych, Resnick, and Davatzikos (2011, 2012); other recent approaches cope with the heterogeneity of subjects using multiplex biomarkers techniques (Steiner, Guest, Rahmoune, & Martins-de-Souza, 2017) and combinations of imaging and genetic patterns (Varol, Sotiras, Davatzikos, & Alzheimer’s Disease Neuroimaging Initiative, 2017), while a strategy to overcome intersubject variability while predicting behavioral variables from imaging data has been proposed in Takerkart, Auzias, Thirion, and Ralaivola (2014). Connectivity features have been used in data-driven approaches for analysis and classification of MRI data in Amico et al. (2017) and Iraji et al. (2016). The purpose of this work is to introduce a novel approach that is rooted on the notion of *consensus* clustering (Lancichinetti & Fortunato, 2012), a strategy developed for community detection in complex networks (Barabási, 2003).

To introduce our method, let us assume that a connectivity matrix is associated with each item to be classified (usually a subject, but also individual scans for the same subject as in the example illustrated below). The goal of supervised analysis is to mine those features of matrices that provide the best prediction of available environmental and phenotypic factors, such as task performance, psychological traits, and disease states. When it comes to using unsupervised analysis of matrices to find groups of subjects, the most straightforward approach would be to extract a vector of features from each connectivity matrix, and to cluster these vectors using one of the commonly used clustering algorithms. The purpose of the present work is to propose a new strategy for unsupervised clustering of connectivity matrices. In the proposed approach the different features, extracted from connectivity matrices, are not combined in a single vector to feed the clustering algorithm; rather, the information coming from the various features are combined by constructing a *consensus* network (Lancichinetti & Fortunato, 2012). Consensus clustering is commonly used to generate stable results out of a set of partitions delivered by different clustering algorithms (and/or parameters) applied to the same data (Strehl & Ghosh, 2002); here, instead, we use the consensus strategy to combine the information about the data structure arising from different features so as to summarize them in a single consensus matrix.

**Figure 1.**
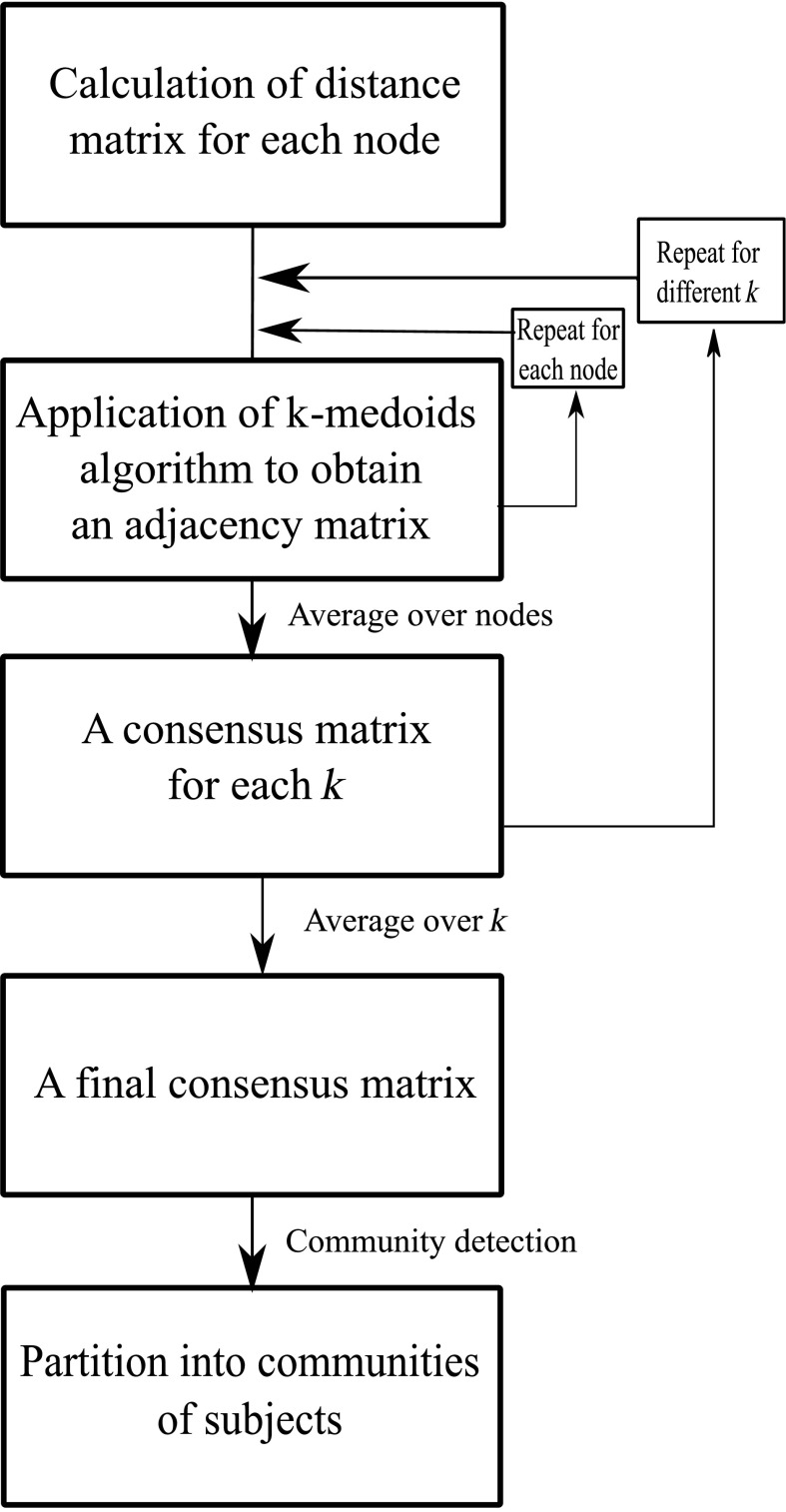
Flowchart of the proposed methodology.

The unsupervised strategy that we propose here to group subjects, without using phenotypic measures, can be summarized as follows, and as depicted in Figure 1: (a) define, for each node, a distance matrix for the set of subjects; (b) cluster the distance matrix for each node; (c) build the consensus network from the corresponding partitions; and (d) extract groups of subjects by finding the communities of the consensus network thus obtained. We remark that the proposed approach provides not only a partition of subjects in communities, but also the consensus matrix, which is a geometrical representation of the set of subjects. In the next section we describe in detail the method and apply it to a toy model, then we show the application on two real MRI datasets. Finally, some conclusions are drawn.

## METHODS

Let us consider *m* subjects whose functional (structural) *N* × *N* connectivity matrix (Rubinov & Sporns, 2010), where *N* is the number of nodes, will be denoted by {**A** (**i** **,** **j**)_*α*_}, *α* = 1, … , *m* and *i*, *j* = 1, … , *N*. For each node *i*, we build a distance matrix for the set of subjects as follows. Consider a pair of subjects *α* and *β*, and consider the corresponding nodal connectivity patterns {**A** (**i** **,** **:**)_*α*_} and {**A** (**i** **,** **:**)_*β*_}; let *r* be their Spearman correlation. As the distance between the two subjects, for the node *i*, we take *d*_*αβ*_ = 1 − *r*; other choices for the distance can be used, like, for example $d_{\alpha \beta }=\sqrt{2(1-r)}$, where *r* is the Pearson correlation. The *m* × *m* distance matrix *d*_*αβ*_ corresponding to node *i* will be denoted by **D**_*i*_, with *i* = 1, … , *N*. The set of *D* matrices may be seen as corresponding to layers of a multilayer network (Boccaletti et al., 2014), each brain node providing a layer.

Each distance matrix **D**_*i*_ is then partitioned into *k* groups of subjects using k-medoids method (Brito, Bertrand, Cucumel, & Carvalho, 2007). Subsequently, an *m* × *m* consensus matrix *C* is evaluated: its entry *C*_*αβ*_ indicates the number of partitions in which subjects *α* and *β* are assigned to the same group, divided by the number of partitions *N*. The number of clusters *k* may be kept fixed, thus rendering the consensus matrix depending on *k*; a better strategy, however, is to average the consensus matrix over *k* ranging in an interval, so as to fuse, in the consensus matrix, information about structures at different resolutions.

The consensus matrix, obtained as explained before, is eventually partitioned in communities by modularity maximization, with the consensus matrix *C* being compared against the ensemble of all consensus matrices one may obtain randomly and independently permuting the cluster labels obtained after applying the k-medoids algorithm to each of the set of distance matrices. More precisely, a modularity matrix is evaluated as

B=C−P,

where *P* is the expected coassignment matrix, uniform as a consequence of the null ensemble chosen here, obtained by repeating many times the permutation of labels; the modularity matrix *B* is eventually submitted to a modularity optimization algorithm to obtain the output partition by the proposed approach. We used the community Louvain routine in the Brain Connectivity Toolbox (Rubinov & Sporns, 2010), which admits modularity matrices instead of connectivity matrices as input.

We remark that the proposed approach has similarities with the one adopted in Shehzad et al. (2014), where techniques from genome-wide association studies coping with the problem of a huge number of comparisons were applied to connectomes, thus identifying nodes whose whole-brain connectivity patterns vary significantly with a phenotypic variable. The approach in Shehzad et al. (2014) consists of two steps. First, for each node in the connectome, a whole-brain functional connectivity map is evaluated, and then the similarity between the connectivity maps of all possible pairings of participants, using spatial correlation, is calculated. Then, in the second stage, a statistic is evaluated for each node, indicating the strength of the relationship between a phenotypic measure and variations in its connectivity patterns across subjects. The main similarity with the proposed approach is that in both methods, for each node in the connectome, the comparison between the connectivity maps yields a distance matrix in the space of subjects.

## A TOY MODEL

As a toy model to describe the application of our method, we simulate a set of 100 subjects, divided in four groups of 25 each. The subjects are supposed to be described by 30 nodes. We will compare our proposed approach with a standard procedure such as averaging the distance matrices and then applying the clustering algorithm to the average distance matrix.

The distance matrices corresponding to the first 10 nodes are constructed in the following way: the distance for pairs belonging to the same group is sampled uniformly in the interval [0.1, 0.4], while the distance for pairs belonging to different groups is sampled uniformly in the interval [0.2, 0.4]. The distance matrices corresponding to the 20 remaining nodes have all the entries sampled uniformly in the interval [0.2, 0.4]. It follows that in our toy model only 10 nodes, out of 30, carry information about the presence of the four groups.

First of all, we evaluate the distance matrix among subjects, averaged over the 30 nodes, and apply the k-medoids algorithm to this matrix , searching for *k* = 4 clusters (thus exploiting the knowledge of the number of classes present in data); this procedure leads to an accuracy of 0.89, measured as follows. Let us call {*G*_*α*_}, *α* = 1, … , 4 the four groups in the model and let *M* be the minimum between 4 and the number of clusters found by modularity maximization clustering; we denote {*C*_*i*_}, *i* = 1, … , *M* the largest *M* clusters found by clustering. The accuracy is then given by

$$\frac{1}{m}\overset{M}{\underset{i=1}{\sum }}max_{\alpha }|G_{\alpha }\cap C_{i}|,$$

where |*G*_*α*_ ∩ *C*_*i*_| is the cardinality of the intersection of the two sets, and *m* = 100 is the total number of subjects.

**Figure 2.**
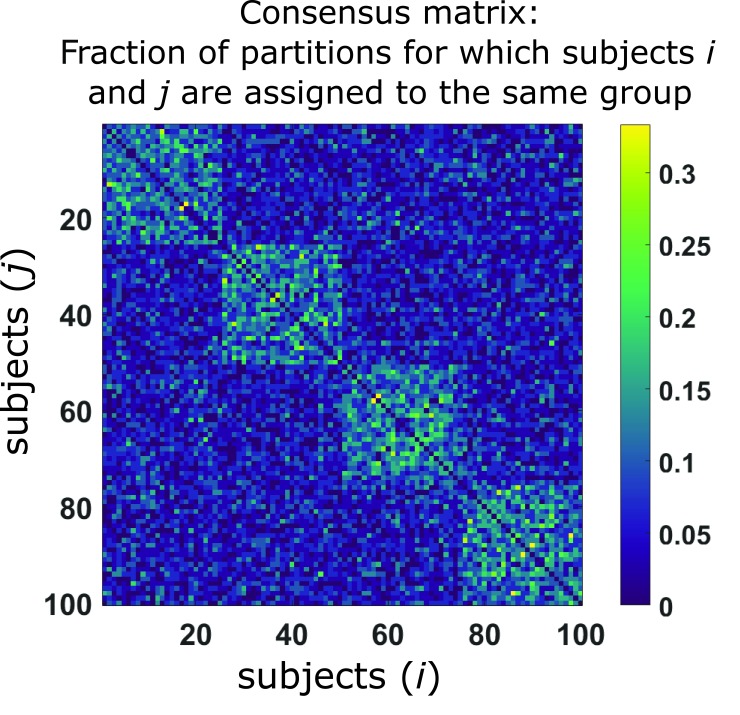
Consensus matrix among subjects in the toy model, obtained by applying k-medoids with *k* = 10 separately to each of the 30 layers. Each entry *C*_*αβ*_ of the matrix represents the number of partitions in which subjects *α* and *β* were assigned to the same group, divided by the number of partitions *N*.

Subsequently, we run the proposed approach by applying separately to each distance matrix for each of the 30 nodes the k-medoids algorithm with varying *k*. We then build the corresponding consensus matrix. For example, in Figure 2 the consensus matrix among subjects is depicted as obtained by applying k-medoids with *k* = 10 separately to each of the 30 layers. Then, the communities of the consensus matrices have been estimated as described in the previous section.

**Figure 3.**
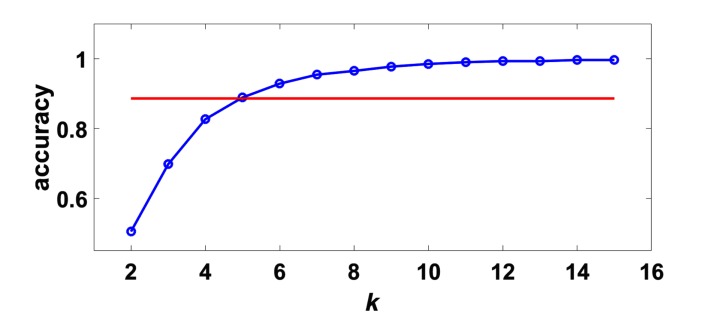
The accuracy of the partition, provided by modularity maximization on the consensus matrix, is depicted versus *k*. The horizontal line represents the accuracy obtained by clustering the average distance matrix using k-medoids and *k* = 4.

In Figure 3 the accuracy of the partition, provided by modularity maximization on the consensus matrix, is depicted versus *k*, in order to show how it varies with *k*: it shows that the proposed method performs better than the partition of the average distance matrix on this example, for large *k*; we remark that the accuracy 0.89 is reached by k-medoids on the average distance using *k* = 4, that is exploiting the knowledge of the number of groups present in the dataset, while the proposed algorithm determines both the number of clusters and the partition. Intuitively, the proposed approach works better in this example for large *k*, because in the distance matrix corresponding to an informative node, due to chance, the block corresponding to a group is seen as fragmented in smaller pieces; those pieces can be retrieved using k-medoids with large *k*. On the other hand, when the consensus is made across the different informative nodes, all those pieces merge in the consensus matrix and build the block corresponding to the four groups.

It is also worth noting that the accuracy by clustering the averaged consensus matrix (over the values of *k*) is one, that is, perfect group reconstruction. Averaging over the values of *k* appears then to be a convenient strategy. Moreover, averaging over values of parame ters is a common strategy for consensus clustering, hence building the consensus matrix while joining several values of *k* is in line with the philosophy of consensus clustering (Lancichinetti & Fortunato, 2012).

**Figure 4.**
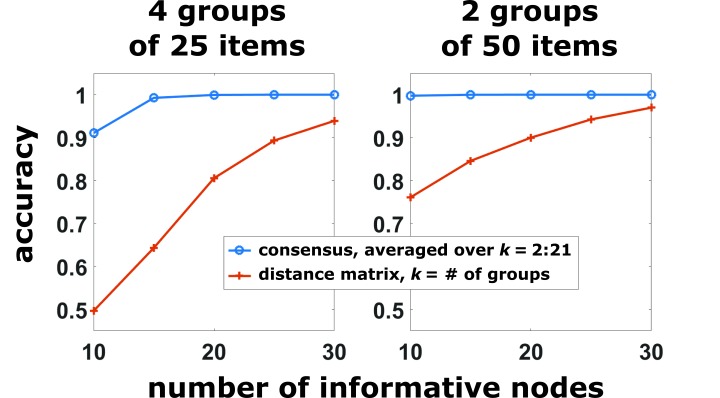
The accuracy of the partition, provided by modularity maximization on the consensus matrix averaged over 20 values of *k*, is depicted versus the number of informative nodes (when it is 30, all the nodes are informative). In the left panel, the plots correspond to four groups of 25 subjects; the blue curve is the accuracy by the proposed method and the red line is the accuracy obtained by clustering the average distance matrix using k-medoids and *k* = 4. In the right panel, the case of two groups, each of 50 subjects, is considered; the blue line is the accuracy by the proposed method and the red line is the accuracy obtained by clustering the average distance matrix using k-medoids and *k* = 2. In all cases the consensus approach gives better results.

In order to show the effectiveness of the proposed approach under different conditions, we change the toy model by varying the number of informative nodes and the number of groups. We also use different parameters with reference to the previous simulations; the distance for pairs belonging to the same group are still sampled uniformly in the interval [0.1, 0.4], while the distance for pairs belonging to different groups is sampled uniformly in the interval [0.15, 0.4]. The results, displayed in Figure 4, show that the proposed approach works better than the application of k-medoids to the average distance matrix.

## APPLICATION TO REAL DATASETS

### Longitudinal dataset

Growing interest is devoted to longitudinal phenotyping in cognitive neuroscience: accord ingly we consider here data from the MyConnectome project (Laumann et al., 2015; Poldrack et al., 2015), where fMRI scans from a single subject were recorded over 18 months. In Shine, Koyejo, & Poldrack (2016), the presence of two distinct temporal states has been identified, which fluctuated over the course of time. These temporal states were associated with distinct patterns of time-resolved blood oxygen level dependent (BOLD) connectivity within individual scanning sessions and also related to significant alterations in global efficiency of brain connectivity as well as differences in self-reported attention. These data were obtained from the OpenfMRI database. Its accession number is ds000031. The functional MRI (fMRI) data were preprocessed with FSL (FMRIB Software Library v5.0). The first 10 volumes were discarded for correction of the magnetic saturation effect. The remaining volumes were corrected for motion, after which slice timing correction was applied to correct for temporal alignment. All voxels were spatially smoothed with a 6 mm FWHM (full width at half maximum) isotropic Gaussian kernel and after intensity normalization, a band pass filter was applied between 0.01 and 0.08 Hz. In addition, linear and quadratic trends were removed. We next regressed out the motion time courses, the average cerebrospinal fluid (CSF) signal, and the average white matter signal. Global signal regression was not performed. Data were transformed to the MNI152 template, such that a given voxel had a volume of 3 mm × 3 mm × 3 mm. Finally, we obtained 268 time series, each corresponding to an anatomical region of interest (ROI), by averaging the voxel signals according to the functional atlas described in Shen, Tokoglu, Papademetris, & Constable (2013).

**Figure 5.**
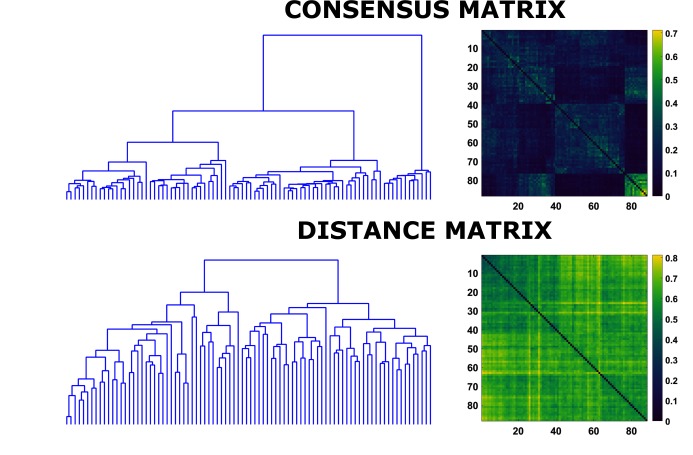
(Top) Concerning the MyConnectome dataset, the consensus matrix, obtained averaging over *k*, by the proposed approach is displayed with nodes ordered according to hierarchical clustering, with the corresponding dendrogram. (Bottom) The average distance matrix, among the different sessions of the same subject, and the corresponding dendrogram.

**Figure 6.**
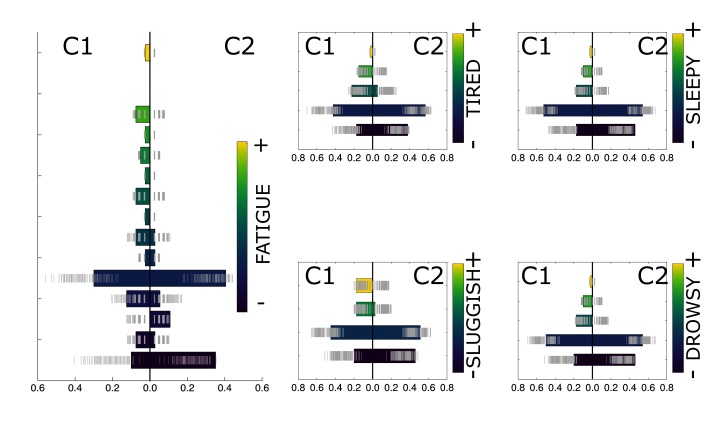
MyConnectome dataset: distributions of the values of the PANAS scores that are sig nificantly different among the two communities found by modularity optimization on the consensus matrix provided by the proposed approach. An expected null distribution, whose quantiles are reported in gray, was obtained by shuffling the association between the PANAS score and connectome matrix.

Each of the 89 sessions resulted in a 268 × 268 matrix of Pearson correlation coefficients. We treated the sessions as if they were connectivity matrices of different subjects, and applied the proposed methodology. In Figure 5 we depict the distance matrix, among the different sessions of the same subject, and the consensus matrix, obtained averaging over 10 values of *k*. Sessions are ordered, in both cases, according to hierarchical clustering; the corresponding dendrograms are also shown in the figure. It is clear that the consensus matrix shows a hier archical structure. Maximization of the modularity provides two communities with modularity equal to 0.175. As depicted in Figure 6, the two communities are significantly different for several PANAS scores, all associated with tiredness. This is assessed visually using a null distribution obtained by shuffling 500 times the pairing between behavioral variable and connectome matrix and with a nonparametric Wilcoxon rank sum test: *drowsy* (Bonferroni cor rected *p* value = 0.028), *tired* (Bonferroni corrected *p* value = 0.041), *sluggish* (Bonferroni corrected *p* value = 0.026), *sleepy* (Bonferroni corrected *p* value = 0.012), *fatigue* (Bonferroni corrected *p* value = 0.022). This confirms the presence of two distinct temporal states. However, the hierarchical structure of the consensus matrix that we obtained suggests that longer longitudinal recordings are needed to further evidence the richness of distinct functional states for single subjects.

**Figure 7.**
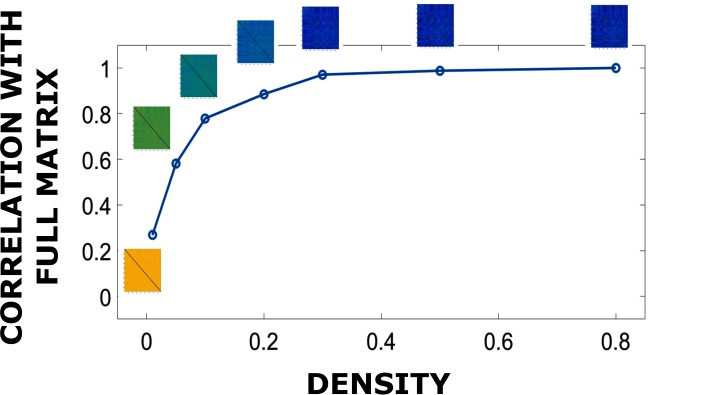
The consensus matrix evaluated by the proposed approach, on the brain connectivity matrices of the MyConnectome dataset, is compared with the consensus matrix from the proposed method on thresholded matrices. The linkwise similarity between the two consensus matrices is evaluated as the Pearson correlation of the corresponding entries in the two matrices, and is plotted versus the density of retained largest entries.

It is also worth considering the effects of network thresholding on the performance of the proposed algorithm: thresholding is a relevant problem in brain connectivity (Fallani, Latora, & Chavez, 2017; Van Wijk, Stam, & Daffertshofer, 2010). The functional networks in this dataset are thresholded so as to retain a varying fraction (density) of the largest entries. In Figure 7 we plot the similarity between the consensus matrices obtained by the proposed algorithm after thresholding and the corresponding consensus matrix in the absence of thresholding, as a function of the density. The similarity between the consensus matrices is evaluated as the Pearson correlation between the entries of the two matrices. On one side the results show the robustness of the proposed approach to moderate thresholding; indeed, up to 20*%* thresholding, the consensus matrix is very close to what is obtained using the full matrices. On the other hand, the consensus matrix by the proposed approach is substantially different for sparser networks. This might speak to the fact that the correlation value is a debatable choice of a thresholding criterion for correlation matrices, and that the proposed approach is suited for weighted networks.

### Resting healthy subjects, functional and structural connectivity

**Figure 8.**
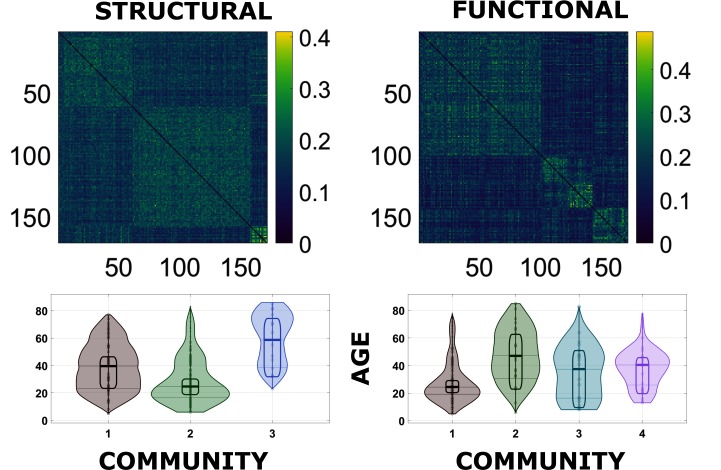
(Top) Concerning the NKI dataset, the consensus matrices found by the proposed approach are shown for structural (top left) and functional (top right) connectivity. (Bottom) The distribution of age values (in years) in the resulting communities are reported. The rectangles indicate the estimator with 95% high density interval, calculated by Bayesian bootstrap. The shaded areas indicate random average shifted histograms, with a kernel density estimate. The code for these plots is available at Pernet (2017).

We consider 171 healthy subjects from the NKI Rockland dataset (Nooner et al., 2012); for each subject we use both the structural diffusion tensor imaging (DTI) network and the functional network, already obtained from processed data as described in Brown, Rudie,Bandrowski, VanHorn, & Bookheimer, 2012. In this case the networks have 118 nodes. In Figure 8 we depict the consensus matrix for both DTI and fMRI networks; modularity maximization yields three communities for DTI networks and four communities for fMRI. Concerning DTI, the three communities are significantly characterized by different ages, with *p* values equal to 9 × 10^−4^, 2 × 10^−5^, and 0.003 for the group comparisons 1-2, 2-3, and 1-3 respectively (see Figure 8). Considering fMRI data, the first group by the proposed algorithm has a different age than the second, the third, and the fourth ones (taken as a whole) with probability 7 × 10^−4^. *P* values reported here refer to a nonparametric rank sum test; similar significance was found using parametric tests. We remark that our method performs different from k-medoids over the average distance, where we obtain two groups with different ages, *t* test with probability 10^−3^ using the functional distance, while no significant difference in age using the structural connectivity.

Inspired by the results found by our method, we also performed a multivariate distance regression (Shehzad et al., 2014), that allowed us to build a pseudo *F* statistic to test whether age correlates with the differences observed in the distance matrix for each node. We have achieved this by comparing the observed *F* statistic with the pseudo *F* distribution (that is, not normal) after 10^5^ data permutations. As expected, for both structural and functional data, we found 124 and 76 nodes statistically related with age respectively, thus suggesting that age is one of the variables responsible for the community structure found by our method.

## CONCLUSIONS

An important issue such as dealing with the heterogeneity that characterizes healthy conditions, as well as diseases, requires the development of effective methods capable of highlighting the structure of sets of subjects at varying resolutions. The approach that we propose here is applied to sets of subjects, each described by a connectivity matrix. We propose a strategy, rooted in complex networks theory, to obtain a consensus matrix that describes the geometry of the dataset, providing at different resolutions groups of similar subjects. While the straight forward application of consensus clustering to a given data set combines the output from different clustering, our proposal is to apply a clustering algorithm separately to the connectivity map of each node. Hence the consensus strategy is exploited to combine the information arising from the different nodes. Obviously, the choice of k-medoids as the clustering algorithm for the individual layers is not mandatory; other algorithms can be used, as well as the definition of the distance among subjects to be used by this algorithm. Moreover, in the present work the features that we considered are the connectivity maps resulting from the whole-brain connectivity pattern of each node; however, other subsets of entries of matrices can be taken as well, and the same strategy can be applied to fuse the different layers and produce a consensus matrix. Likewise, our framework is not limited to considering the whole brain, and therefore it can be applied to analyze specific regions relevant to the problem at hand so as to exploit the benefits of our method. To summarize, our approach aims to disentangle the heterogeneity of groups corresponding to high-level categories, like healthy and diseased, finding natural groups within the cohort of patients (and within the cohort of controls). While dealing with data with both healthy and controls, it can be seen as a preprocessing step, which helps the subsequent construction of a supervised classifier healthy/subject.

## ACKNOWLEDGMENTS

The authors are grateful to Richard Betzel (University of Pennsylvania) and an anonymous referee for valuable suggestions. They also thank Guillaume Rousselet for valuable suggestions on data representation.

## SUPPORTING INFORMATION

The code for the construction of the consensus matrix, out of the set of connectivity matrices, is available at Rasero (2017).

## AUTHOR CONTRIBUTIONS

Javier Rasero: Data curation; Methodology; Software; Writing review & editing. Mario Pellicoro: Investigation; Software; Writing review & editing. Leonardo Angelini: Investigation; Writing review & editing. Jesus M. Cortes: Investigation; Writing review & editing. Daniele Marinazzo: Conceptualization; Data curation; Investigation; Writing review & editing. Sebastiano Stramaglia: Conceptualization; Investigation; Methodology; Supervision; Writing original draft.

## FUNDING INFORMATION

JR acknowledges financial support from the Minister of Education, Language Policy and Culture (Basque government) under the Doctoral Research Staff Improvement Programme.

## TECHNICAL TERMS

Brain connectivity network (connectome): A network in which the nodes are brain regions and the links are anatomical connections (“anatomical/structural connectivity”), or statistical dependencies (“functional connectivity”).
Consensus matrix: Given several partitions of a given set of nodes, for each pair of nodes the consensus matrix provides the fraction of partitions in which the two nodes belong to the same subset.
Distance matrix: For each node, a distance matrix for the set of subjects is constructed based on the Spearman correlation between the nodal connectivity patterns of the given node in the two subjects.
K-medoids: Clustering algorithm similar to k-means, which in contrast chooses data points as center (so-called medoids), making it more robust to outliers.
Resting-state fMRI: Functional magnetic resonance imaging acquired while the subject is simply instructed to stay awake.
PANAS (Positive and Negative Affect Schedule): The PANAS comprises two mood scales, one that measures positive affect and one that measures negative affect. Participants in the PANAS are required to respond to a 20-item test.
